# Supplementary material for: Identification of a Novel Aflatoxin B1-Degrading Strain, Bacillus halotolerans DDC-4, and Its Response Mechanisms to Aflatoxin B1
Source: Toxins (Basel). 2024 May 31;16(6):256. doi: 10.3390/toxins16060256 (PMC11209560; doi:10.3390/toxins16060256)
Supplement: Supplementary file 1 [file toxins-16-00256-s001.zip › toxins-3013979-supplementary.pdf]

Supplementary material: Identification of a Novel Aflatoxin B_1-Degrading Strain, *Bacillus halotolerans* DDC-4, and Its Response Mechanisms to Aflatoxin B_1

Table S1. Statistics of the read alignments in the RNA-Seq study

Sample	Average read length (bp)	Total reads	Total bases (bp)	Q20 (%)	Q30 (%)	GC (%)	N (%)
F1	144.83	32552890	4714622574	98.91%	95.82%	46.90%	0.00%
F2	145.18	28586048	4150103434	99.03%	96.26%	47.37%	0.00%
F3	143.32	34322394	4919246666	99.08%	96.45%	47.59%	0.00%
Y1	145.19	29967602	4350953937	99.11%	96.61%	51.55%	0.00%
Y2	145.04	32955562	4779910823	98.86%	95.66%	46.85%	0.00%
Y3	143.84	24536268	3529366066	99.16%	96.76%	48.69%	0.00%

Samples F and Y represent data for DDC4 untreated and treated by AFB_1, respectively. The numbers (1, 2 and 3) after "-" represent three independent biological replicates for each treatment.

Table S2. The reads mapped to the reference genome

Sample	Total mapped (%)	Mutiple mapped (%)	Uniquely mapped (%)	Reads mapped in proper pairs （%）
F1	95.85%	8.75%	87.10%	83.79%
F2	95.78%	8.91%	86.87%	83.55%
F3	96.29%	9.44%	86.85%	82.87%
Y1	96.12%	6.68%	89.45%	85.58%
Y2	97.56%	7.07%	90.49%	86.77%
Y3	97.05%	9.04%	88.01%	83.28%

Samples F and Y represent data for DDC4 untreated and treated by AFB_1, respectively. The numbers (1, 2 and 3) after “-” represent three independent biological replicates for each treatment.

Table S3. DEGs enriched in developmental process

Gene id	Gene name	Gene description	Related to sporulation	log2 foldchange
RS00170	*SspN*	acid-soluble spore protein SspN	Yes	1.25
RS00990	*DpaB*	dipicolinate synthase subunit B	Yes	2.23
RS00995	*DpaA*	dipicolinic acid synthetase subunit A	Yes	2.41
RS01705	*SigG*	RNA polymerase sporulation sigma factor SigG	Yes	1.47
RS01715	*SpoIIGA*	sigma-E processing peptidase SpoIIGA	Yes	1.59
RS02435	*SplB*	spore photoproduct lyase	Yes	1.10
RS02530	RS02530	glycosyltransferase	Yes	1.32
RS03665	RS03665	DUF1360 domain-containing protein	Yes	1.24
RS04195	*AsnB*	asparagine synthase (glutamine-hydrolyzing)	Yes	1.63
RS04925	*SpoVR*	stage V sporulation protein SpoVR	Yes	1.22
RS05055	RS05055	YhcN/YlaJ family sporulation lipoprotein	Yes	2.07
RS05420	RS05420	gamma type acid-soluble spore protein SspE	Yes	1.75
RS05790	*PdaA*	delta-lactam-biosynthetic de-N-acetylase	Yes	1.82
RS07870	RS07870	penicillin-binding protein 3	No	1.76
RS07985	*GlcU*	glucose uptake protein GlcU	Yes	1.95
RS08100	RS08100	Ger(x)C family spore germination protein	No	1.57
RS08105	RS08105	spore germination protein	No	1.33
RS09830	*SpoIIE*	stage II sporulation protein E	Yes	2.26
RS09910	*GlmU*	bifunctional UDP-N-acetylglucosamine diphosphorylase/glucosamine-1-phosphate N-acetyltransferase GlmU	No	1.39
RS09935	RS09935	small, acid-soluble spore protein, alpha/beta type	Yes	2.42
RS09945	*PrtG*	sporulation-specific protease PrtG	Yes	2.09
RS12135	RS12135	RsfA family transcriptional regulator	Yes	3.84
RS12195	RS12195	penicillin-binding protein	No	1.18
RS12445	*RacA*	chromosome-anchoring protein RacA	Yes	1.93
RS15005	*LytH*	L-Ala-D-Glu endopeptidase	Yes	1.12
RS16210	RS16210	cytochrome ubiquinol oxidase subunit II	Yes	1.31
RS16215	RS16215	cytochrome ubiquinol oxidase subunit I	Yes	2.30

Table S4. Primers used in this study

	Primer sequences (5'-3')
16S-F	AGAGTTTGATCCTGGCTCAG
16S-R	GGTTACCTTGTTACGACTT
RS11000-F	CGGTTTGGTCGATGTGC
RS11000-R	TTTCCGGCCAGTAATCC
RS07845-F	ATTATATGAAACCAGGCAGCG
RS07845-R	GCTACCGCATTTACACGGA
RS04140-F	CAAGTTTCTCCGGAGTTCCA
RS04140-R	GTGTCGGCACCTTGATTTC
internal control-F	GACAGAACAAAGGGCAGC
internal control-R	GGCATGCTGATCCGCGA

Table S5. The concentration and quality of RNA

Sample	The concentration of RNA (ng/μL)	A260/280	A260/230
F1	249.9	2.004	1.804
F2	230.2	1.980	1.812
F3	225.4	2.015	1.821
Y1	172.6	2.062	1.806
Y2	197.9	2.023	1.814
Y3	168.2	1.987	1.807

Samples F and Y represent data for DDC4 untreated and treated by AFB_1, respectively. The numbers (1, 2 and 3) after "-" represent three independent biological replicates for each treatment.

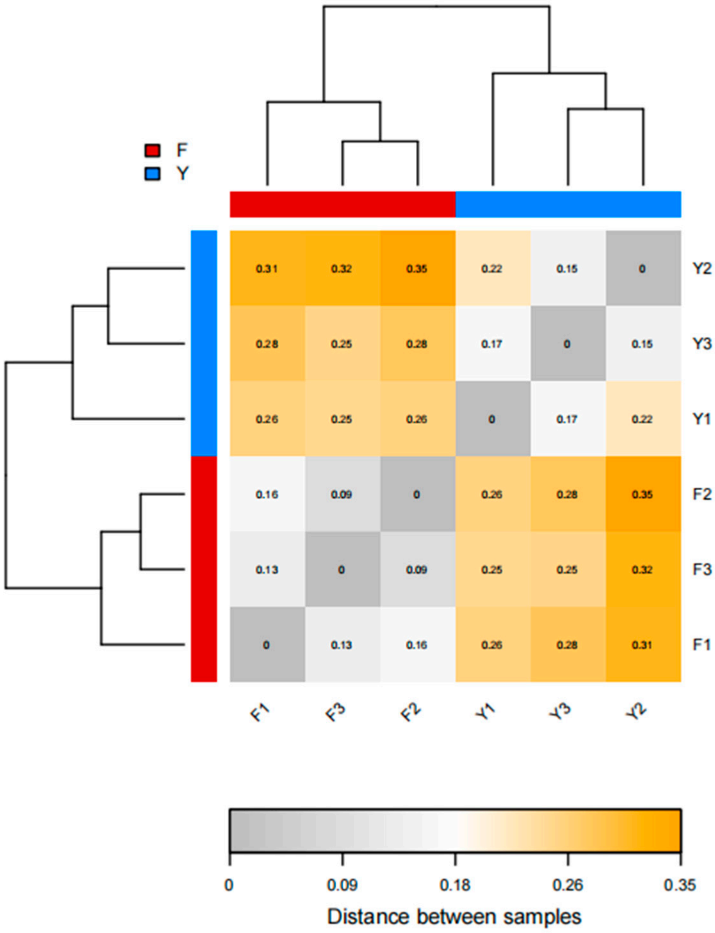

Figure S1. Heatmap of distance between samples

Samples F and Y represent data for DDC4 untreated and treated by AFB1, respectively.

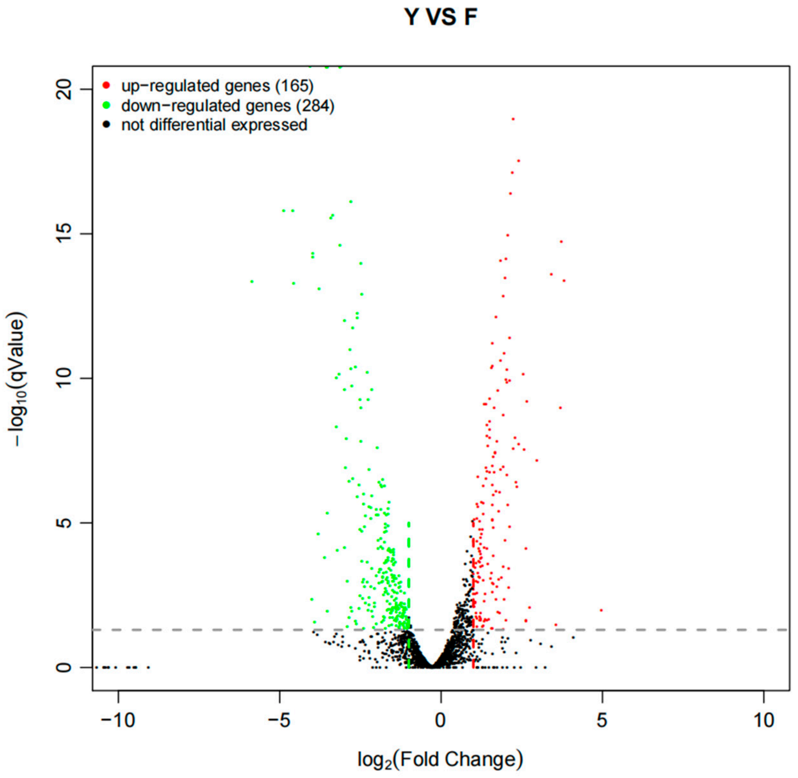

Figure S2. Volcano plot of DEGs

Samples F and Y represent data for DDC4 untreated and treated by AFB1, respectively. Each point represents a gene, with red indicating up-regulated DEGs, green indicating down-regulated DEGs, and black indicating not differential expressed genes.

(A)

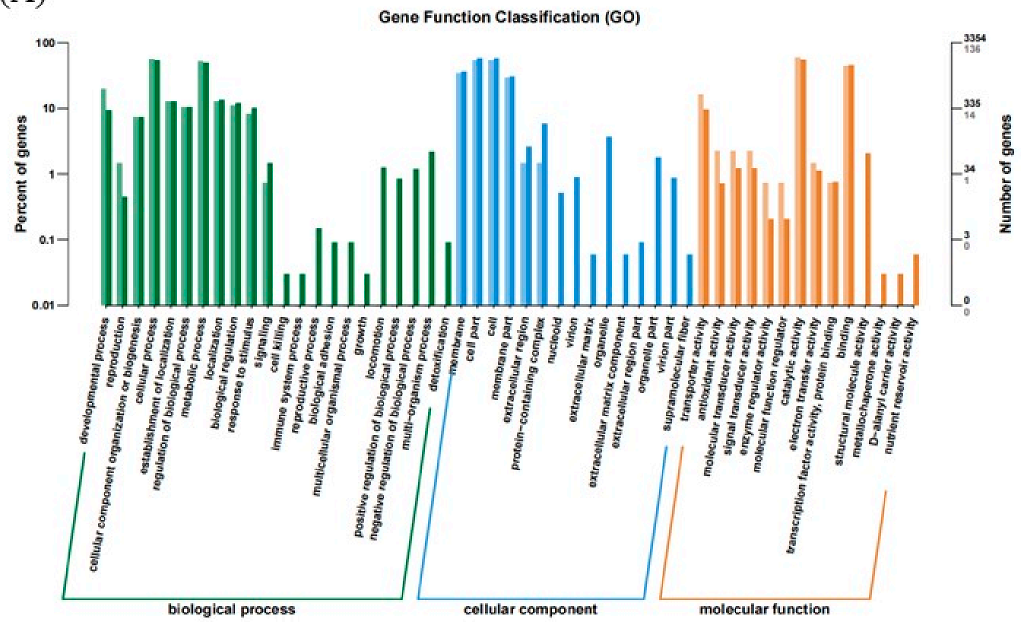

(B)

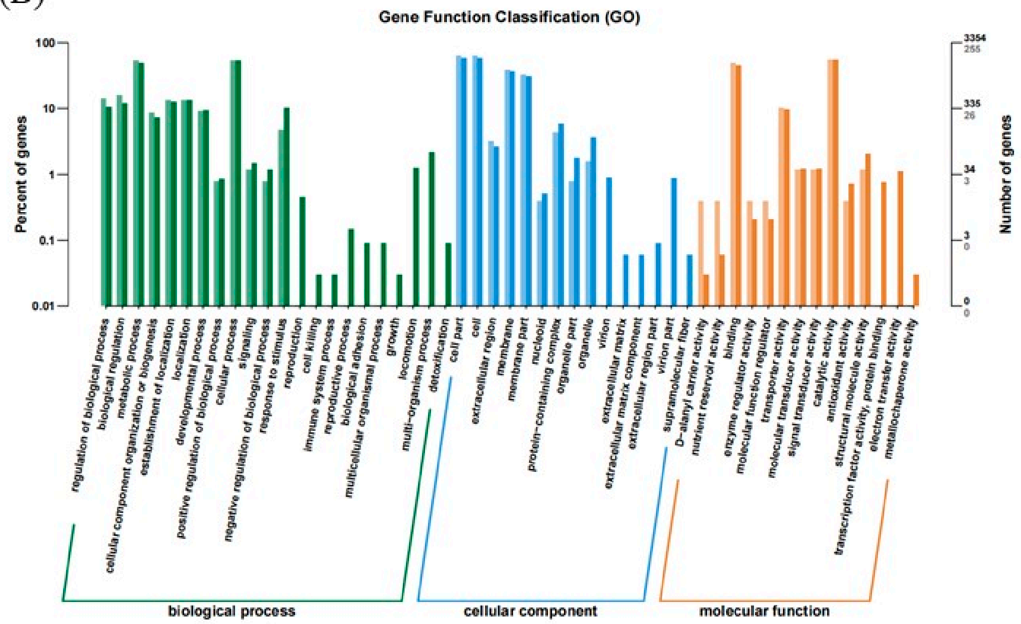

Figure S3. GO term enrichment classification of DEGs

(A) up-regulated DEGs; (B) down-regulated DEGs

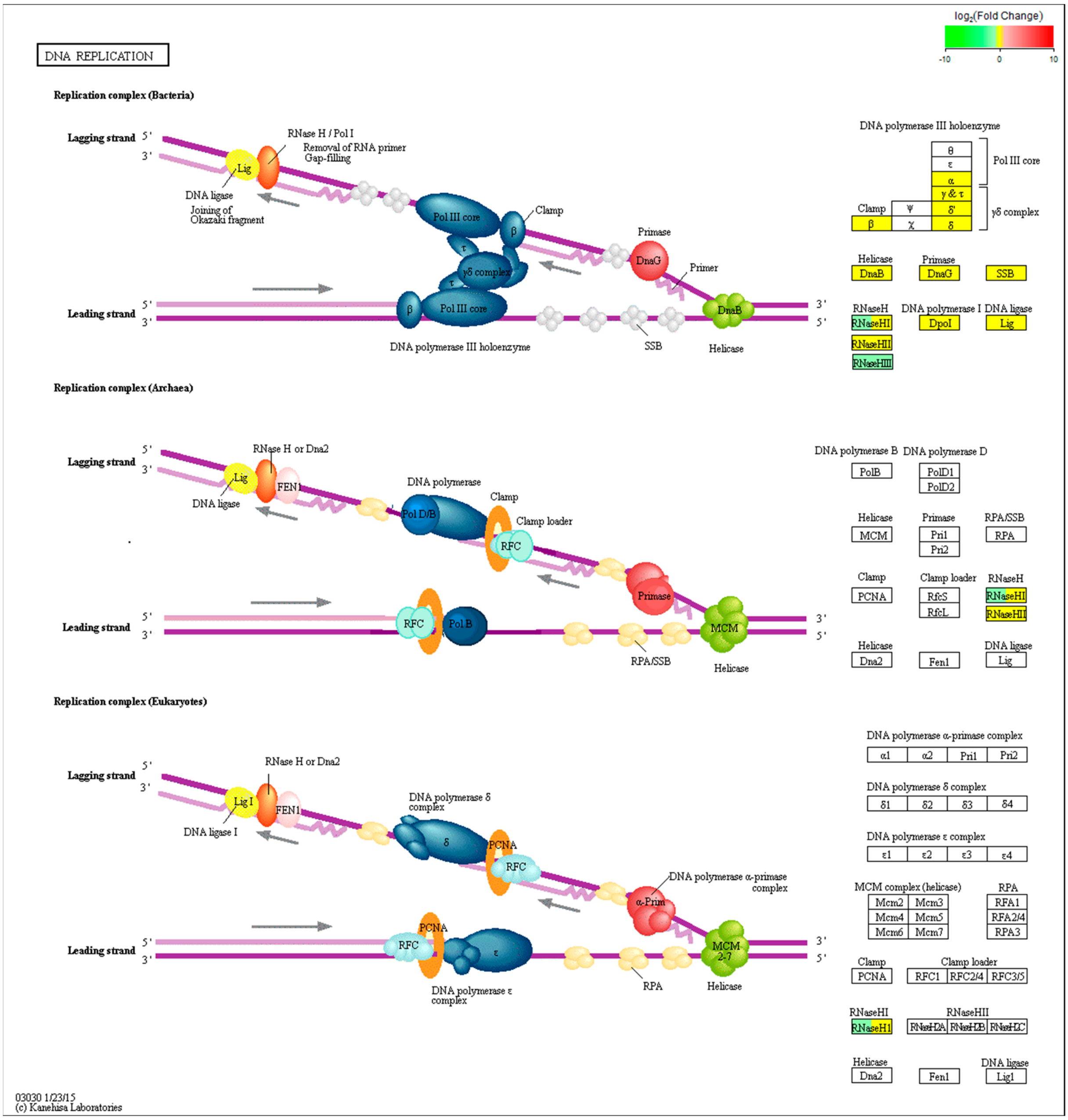

Figure S4. DNA replication
